# Investigation of *Pleurotus Ostreatus* Mushroom-Based Feed Supplementations on Growth Performance and Immune Function in Male Japanese Quails (*Coturnix Japonica*)

**DOI:** 10.3390/vetsci13050496

**Published:** 2026-05-20

**Authors:** Gréta Törős, Gabriella Gulyás, Renáta Knop, Csaba Szabó, Gebrehaweria K. Reda, Sawadi F. Ndunguru, Ducza László, Ágoston Botond Gaál, József Prokisch, Levente Czeglédi

**Affiliations:** 1 Institute of Animal Science, Biotechnology and Nature Conservation, Faculty of Agricultural and Food Sciences and Environmental Management, University of Debrecen, Böszörményi Street 138, 4032 Debrecen, Hungary; gulyas@agr.unideb.hu (G.G.); dr.knop.renata@agr.unideb.hu (R.K.); szabo.csaba@agr.unideb.hu (C.S.); gebrek2000@gmail.com (G.K.R.); sawadindunguru@gmail.com (S.F.N.); jprokisch@agr.unideb.hu (J.P.); czegledi@agr.unideb.hu (L.C.); 2 Doctoral School of Animal Science, Faculty of Agricultural and Food Sciences and Environmental Management, University of Debrecen, Böszörményi Street 138, 4032 Debrecen, Hungary; 3 Department of Anatomy, Histology and Embryology, Faculty of Medicine, University of Debrecen, 4032 Debrecen, Hungary; ducza.laszlo@anat.med.unideb.hu (D.L.); gaal.botond@med.unideb.hu (Á.B.G.)

**Keywords:** antioxidant, beta-glucan, Japanese quail, growth performance, immune system activity, oyster mushroom

## Abstract

Growing concern over antibiotic resistance in poultry production has increased interest in natural feed additives such as *Pleurotus ostreatus* (oyster mushroom)-derived products. This study evaluated three freeze-dried oyster mushroom powders: total fresh fruiting body (OMP-TF), cooked solid residue (OMP-CSR), and cooked liquid fraction (OMP-CL), as dietary supplements in male *Coturnix japonica*. A β-glucan positive control and basal diet negative control were included. Birds (1-day-old) were fed experimental diets from 1 to 28 days. An *Escherichia coli* lipopolysaccharide (LPS) challenge was applied 12 h before sampling to induce an immune system activity. Growth performance, immune system activity, spleen relative weight, ileum morphometry, and antioxidant capacity were then evaluated to determine the effects of the experimental diets. Mushroom supplementation significantly modulated body weight during weeks 2–3 and overall weight gain, with OMP-TF maintaining higher body weight at week 4. Relative spleen weight, villus morphometry, cytokine responses (IL-1β, IL-2, IL-4), and total antioxidant capacity were not significantly altered compared with LPS controls. Overall, *P. ostreatus* supplementation modulated growth performance under inflammatory challenge conditions, without significantly affecting the measured immune or intestinal parameters. These findings suggest a potential role of mushroom-derived products as growth-supporting feed additives; however, further studies are needed to clarify their effects on immune function and intestinal health.

## Simple Summary

The use of antibiotic growth promoters in poultry production is increasingly restricted, creating demand for natural alternatives to support growth and health. Oyster mushroom (*Pleurotus ostreatus*) contains bioactive compounds, including β-glucans, with potential immunomodulatory and growth-promoting effects. This study evaluated three freeze-dried oyster mushroom preparations (from the total fruiting body, cooked solid residue, and cooked liquid fraction) as dietary supplements in male Japanese quails (*Coturnix japonica*) under *Escherichia coli* lipopolysaccharide (LPS)-induced inflammatory challenge. Growth performance, immune-related cytokines, spleen weight, intestinal morphology, and antioxidant capacity were assessed over a 28-day feeding period. Mushroom supplementation improved body weight and body weight gain, particularly during the rapid growth phase, with the strongest and most persistent effect observed in birds receiving the total fruiting body preparation. However, no significant effects were detected on cytokine levels, spleen weight, intestinal morphology, or antioxidant capacity compared with LPS controls. Overall, *P. ostreatus* supplementation showed growth-supporting potential under inflammatory conditions, but its immunological and physiological mechanisms require further investigation.

## 1. Introduction

Since ancient times, mushrooms have been widely used in traditional medicine to combat various diseases. In recent years, they have gained significant attention as natural, high-safety functional feed supplements with potential antibiotic growth-promoting properties in poultry farming [1,2,3]. This interest is driven by global concerns over antibiotic resistance, which has emerged due to the overuse and inappropriate application of antibiotics in livestock production [4,5]. Excessive reliance on antibiotics has led to the proliferation of resistant pathogens, compromising their effectiveness in poultry and human medicine. The transfer of resistant bacterial strains from animals to humans through the food chain has intensified the public health crisis, leading to restrictions and outright bans on antibiotic growth promoters in many regions [6,7]. This has necessitated the search for alternative solutions to maintain poultry health and productivity without relying on conventional antibiotics.

Scientific studies employing diverse methodologies have increasingly highlighted the therapeutic benefits of mushroom-derived products, particularly their potential as non-antibiotic growth promoters in poultry farming [8]. Among the various mushrooms explored, *Pleurotus ostreatus* (oyster mushroom) stands out due to its broad-spectrum antimicrobial properties, prebiotic potential, and positive effects on gut microbiota. These properties collectively contribute to immune modulation, enhancing poultry health and resilience against infections [7,9]. Additionally, oyster mushrooms possess potent antioxidant properties, neutralizing free radicals and reducing oxidative stress, two factors implicated in poultry diseases [10,11]. The poultry industry has shown a growing interest in replacing synthetic antioxidants with natural alternatives like edible mushroom-derived compounds, aligning with consumer preferences for residue-free and health-safe products. Moreover, oyster mushroom cultivation offers environmental benefits by repurposing agricultural byproducts, thereby contributing to sustainability [12,13]. This makes oyster mushrooms a health-promoting agent and an ecologically significant resource.

One of the key bioactive compounds in oyster mushrooms is β-glucan, a polysaccharide known for its immunomodulatory properties. β-glucans are crucial in enhancing immune function and helping poultry combat antibiotic-resistant infections [14,15,16,17,18]. This has led to the widespread adoption of *P. ostreatus* (oyster mushroom)-based feed supplements in poultry nutrition. However, while yeast-derived β-glucans have been extensively studied, further comparative research is needed to evaluate the efficacy of mushroom-derived β-glucans in poultry. β-glucans derived from *P. ostreatus* are primarily β-(1→3)/(1→6)-linked polysaccharides, which are recognized for their potent immunomodulatory properties compared with cereal-derived β-glucans [19,20,21].

The biological activity of β-glucans and other bioactive compounds in *P. ostreatus* can vary significantly depending on the processing method. Different preparation techniques, such as freeze-drying, cooking, and liquid extraction, may influence these compounds’ structural integrity and bioavailability, ultimately affecting their physiological impact [22,23,24,25]. Despite the known immunostimulatory effects of *P. ostreatus*, limited research has explored how processing methods alter its bioactive properties and influence poultry health outcomes.

This study evaluated the effects of freeze-dried *Pleurotus ostreatus* powder prepared using three different processing methods on growth performance, immune system activity, and health status of male Japanese quails (*Coturnix japonica*). We hypothesized that dietary inclusion of oyster mushroom powder may enhance immune function and contribute to modulated health outcomes, offering a potential natural alternative to antibiotic use in poultry production.

## 2. Materials and Methods

### 2.1. Oyster Mushroom Product Preparation Technique

We have previously introduced and thoroughly characterized the manufacturing process of the mushroom powders used in the present study. Importantly, the same production batches that were characterized in our earlier work [26] were used directly in the current feeding experiment without any further modification. Specifically, the total β-glucan content, the primary immunomodulatory component was found to be 36.25 ± 0.50 *w*/*w*% for OMP-TF, 37.95 ± 0.78 *w*/*w*% for OMP-CSR, and 20.27 ± 0.58 *w*/*w*% for OMP-CL. This characterization provides the biochemical basis for evaluating the physiological responses observed in the Japanese quail model.

**Figure 1:**
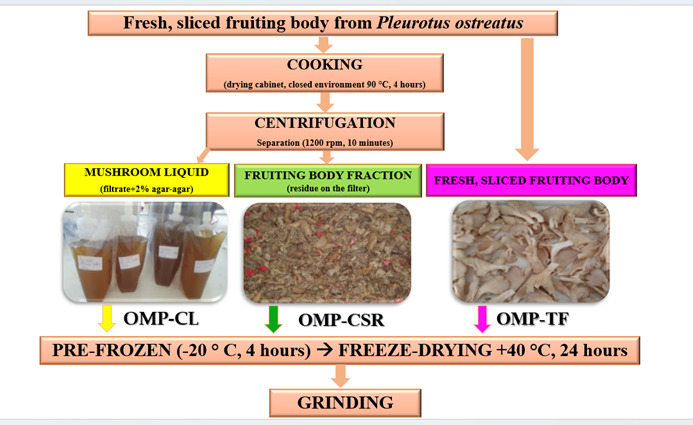
The schematic figure for the manufacturing process of mushroom powders. Freeze-dried oyster mushroom powder from cooked mushroom liquid-filtrate (OMP-CL); Freeze-dried oyster mushroom powder from the cooked fruiting body: residue on the filter (OMP-CSR). Freeze-dried oyster mushroom powder from the total fresh fruiting body (OMP-TF).

Figure 1 presents a detailed illustration of the step-by-step manufacturing process, showing the raw materials and their transformation into the final mushroom powders (OMP-TF, OMP-CSR, and OMP-CL), which were identical to those used in the present trial.

**(A)**  **OMP-TF** → Fresh *P. ostreatus* mushrooms were washed and quartered.
**(B)**  **OMP-CL** → After cooking the fresh, quartered OMPs at 90 °C (4 h) in a sealed pressure cooker, the mushrooms were centrifuged and filtered to produce a liquid extract. The liquid was gelled with 2% agar-agar.
**(C)**  **OMP-CSR** → The cooked fruiting body with reduced (centrifugated) liquid content has also been administered for further manufacturing processes (freeze-drying and grinding) to prepare a consistent and stable formula.

All of the samples (OMP-CL, OMP-CSR, OMP-TF) were pre-frozen (−20 °C, 4 h), then freeze-dried with a shelf (plate) temperature of 40 °C under vacuum conditions for 24 h. The resulting lyophilized materials were ground to obtain a dry powder and stored in airtight containers at temperatures below 25 °C until administration in animal experiments.

### 2.2. Animal Experiment

**Figure 2:**
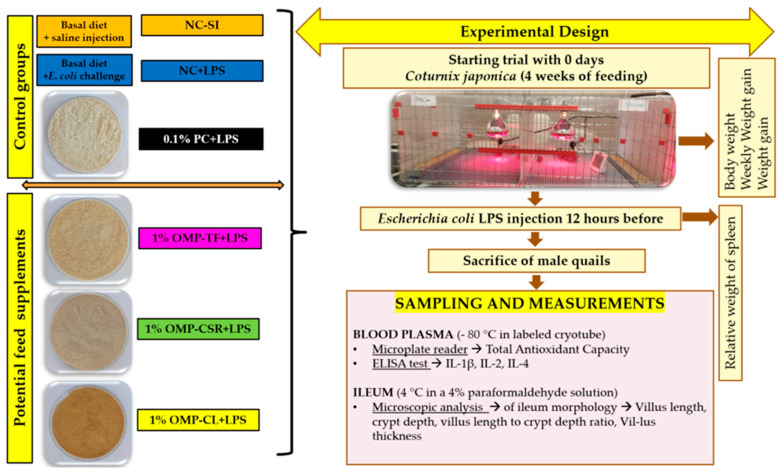
Schematic figure for the poultry experiments’ (4-week feeding trial) introduction of experimental design (treatments with pictures, test methods, and parameters).

Figure 2 below illustrates the experimental setup for the poultry study, detailing six groups containing oyster mushroom-based supplements, a positive and a negative and a placebo control groups, the age of the starter animal models, the gender applied for sample collection, and the types of equipment used to perform the experiments (including tested parameters).

### 2.3. Experimental Animals and Housing

Animal experiments were conducted in October 2024, and the mushroom powders were prepared immediately prior to the experiment. Our study uses male Japanese quail (*Coturnix japonica*) models. Due to its small size, rapid growth, high reproductive rate, and disease resistance, the Japanese quail plays a significant role in the poultry industry. Additionally, it is widely used as a model organism in scientific research because of its genetic simplicity, ease of handling, and adaptability to controlled environments [27,28].

Budai Quail Farm (Hungary) provided the Japanese quail eggs required for the experiment. They were incubated and hatched under controlled conditions at the Animal House of the Institute of Animal Science, Biotechnology, and Nature Conservation of the University of Debrecen (Hungary), where the animal experiments were also performed. R-COM Maru 190 digital incubator were used to hatch Japanese quail eggs under a constant temperature of 37.8 ± 0.5 °C and 50–60% relative humidity.

The feeding trial was conducted using a completely randomized design with six dietary treatments and two replicate cages per treatment. This distinction reflects the hierarchical structure of the experimental design. Each cage contained 10 one-day-old Japanese quail chicks (*Coturnix japonica*), which were individually identified and monitored throughout the experiment (28 days). The experimental unit for growth performance parameters was the individual bird, whereas the cage served as the experimental unit for feed intake and feed conversion ratio. In total, 120 quails were used in the experiment (6 treatments × 2 replicate cages × 10 birds per cage). Since birds were housed as mixed-sex groups until 21 days of age, feed intake and feed conversion ratio data collected during this period represent cage-based measurements from mixed-sex populations.

Sex determination was performed at 21 days of age because reliable sexing of Japanese quail is not possible at hatch. Sexual dimorphism based on plumage coloration and cloacal gland development becomes evident only around 3 weeks of age [29]. Therefore, birds were reared as a mixed-sex population until accurate sex identification could be achieved.

After gender identification, male quails were selected and identified through a leg ring, then selected for LPS injection and sampling as visible at (2.5). The different groups were marked with distinct colors, and each sample was assigned a unique identification number. Only male *C. japonica* were included in the final analyses to ensure physiological uniformity in growth and immune system activities, thereby reducing variability associated with sex-dependent differences. The graphs presenting the results use the same color coding as summarized in Table 1.

Birds were housed in cages (38.85 cm^2^ per bird) under controlled conditions (started with 35 ± 3 °C temperature (until the first week), and reducing it weekly with 3 °C, and with 60–75% relative humidity. During the brooding period (0–3 weeks), chicks received 23–24 h of light/day initially, gradually decreasing, at 30–40 lux to promote feeding and uniform growth, while during the grower period (3–4 weeks), they were exposed to 16–18 h of light at 10–15 lux to support steady growth and reduce stress, with additional UVB lamps used for the chicks.

### 2.4. Dietary Treatments and E. coli LPS Injection

Different freeze-dried mushroom powder preparations have been tested, each incorporated into the feed at a 1% concentration (Figure 2). Additionally, the positive control group received a supplement containing 1,3/1,6 β-glucan from *Saccharomyces cerevisiae* in 0.1% inclusion rate and with 80 *w*/*w*% of β-glucan content (Medinvest Hungary). The inclusion level of 0.1% β-glucan was selected based on previous studies demonstrating its immunomodulatory efficacy in poultry, particularly under inflammatory challenge conditions, without adverse effects on growth performance [30,31].

The basal diet was prepared on a corn-soybean-wheat basis according to NRC (1994) [2] as described in Table 2, where β-glucan and mushroom-derived products were included as functional feed additives, and their contribution to the calculated nutrient composition was not considered. Diets were formulated based on the major ingredients to meet or exceed NRC (1994) requirements.

To induce an immune system activity, male quails were transferred to separate cages 12 h before termination sampling and intraperitoneally injected with *E. coli* lipopolysaccharide (LPS) at a dose of 1.5 mg/kg body weight. The O55:B5 serotype LPS (L2880, Sigma, St. Louis, MO, USA) was dissolved in sterile isotonic saline to obtain a final concentration of 1.5 mg/mL, and the injection volume was adjusted according to individual body weight. LPS are large molecules composed of lipids and polysaccharides, acting as bacterial toxins. They consist of an O-antigen, an outer core, and an inner core, covalently linked and present in the outer membrane of Gram-negative bacteria. LPS administration is widely used to induce an acute immune system activity, leading to the activation of immune cells and functional stimulation of immune organs such as the spleen [32].

The “placebo group” receiving a saline injection (NC+SI) was administered sterile saline at the same injection volume to control for the effects of the injection procedure itself. This control allowed differentiation between responses induced by LPS and those associated with the injection process.

Japanese quails were housed under standard conditions with ad libitum access to feed and water, monitored at least twice daily for health and signs of distress, with humane endpoints established a priori (significant weight loss, severe lethargy, or inability to access feed/water), and no unexpected adverse events occurred during the study.

### 2.5. Sampling

After four weeks (28 days), male birds from each experimental group were identified based on breast feather color, leg bands, and cloacal examination. Male birds (n = 6/group) were then humanely euthanized by cervical dislocation performed by a veterinary professional.

A robust traceability system was employed to ensure accurate monitoring of weight gain and precision in all experimental measurements. Prior to the euthanasia of the male *C. japonica*, the final live body weight was recorded. Following slaughter and dissection, the spleen weight was measured. All collected data were systematically documented using the birds’ corresponding identification numbers.

After termination, plasma and ileum samples were collected and stored under appropriate conditions for later analysis (Figure 2):

(1.) For morphological examination → 1 cm ileum segments were immediately collected. These samples were preserved in a 4% paraformaldehyde solution (Sigma-Aldrich, St. Louis, MO, USA) and stored at room temperature.
(2.) For immune system activity, biochemical analysis, and total antioxidant capacity determination → Blood samples were collected into ethylenediaminetetraacetic acid (EDTA) tubes and centrifuged at 3000× *g* for 10 min. After separation, the plasma was transferred into labeled cryotubes and stored at −80 °C in an ultra-deep freezer (VWR International Hungary Kft., Debrecen, Hungary). The frozen plasma samples were later used for laboratory analyses.

### 2.6. Laboratory Measurements

#### 2.6.1. Growth Performance and Relative Weight of Spleen

On day 1, chicks were weighed as a group prior to individual identification in order to minimize handling stress during the immediate post-hatch period. Birds were subsequently randomly allocated to experimental treatments to ensure homogeneous initial body weights among groups. Individual identification using leg rings was applied after stabilization, and individual body weight measurements were recorded from the first week onward.

Body weight (BW) was recorded weekly, and weekly body weight gain (BWG) as well as body weight gain over the total experimental period were calculated using all male chicks in each treatment group. Body weight was measured using a digital scale with a precision of ±0.01 g. On day 1, chicks were weighed as a group without leg rings or individual identification. Although body weight was measured repeatedly, week-wise comparisons were performed to evaluate temporal treatment effects, which is commonly applied in poultry growth studies under similar experimental conditions.

Thereafter, individual body weight was measured weekly in all chicks fitted with leg rings and identification numbers on day 7 (week 1), day 14 (week 2), day 21 (week 3), and day 28 (week 4) of the experiment.

Weekly body weight gain was calculated to evaluate growth dynamics during the 4-week trial. For each bird, weekly BWG was determined as the difference between body weight measured at the end of a given week and that recorded at the end of the previous week, according to the following formula:

*Weeklyweight gain* = *Week_i_* − *Week_i−_*_1_

Body weight gain for the total experimental period was calculated as the difference between body weight at day 28 and body weight at day 0.

Spleen weight was measured immediately after euthanization to a precision of ±0.01 g. and relative spleen weight was calculated by dividing the respective final body weight of each bird. The equation, as described by Sławińska (2014) [33] is:

*Relative spleen weight* = (*Spleen Weight* (g)/*Live Weight* (g)) × 100.

#### 2.6.2. Interleukin Levels

The levels of interleukin (IL-1β, IL-2, IL-4) in plasma samples were determined using ELISA (n = 6 per treatment) according to the manufacturer’s instructions (ECH0040, ECH0041, ECH0044, Wuhan Fine Biotech Co., Ltd., Wuhan, China). These interleukins were selected to represent key components of the immune system activity in response to inflammatory stimulation. IL-1β is a principal pro-inflammatory cytokine involved in the acute-phase response, IL-2 is a key regulator of T-cell proliferation and adaptive immunity, and IL-4 is associated with the modulation of Th2-type immune system activities and anti-inflammatory regulation.

Samples and standards were measured in duplicate. Absorbance was measured using a Synergy HTX Multi-Mode microplate reader (BioTek Instruments Inc., Winooski, VT, USA) at 450 nm wavelength. Interleukin concentrations were calculated using the equation from the linear regression of the obtained standard curve. The sensitivity of the assays for interleukin (IL-1β, IL-2, IL-4) was 1.875 pg/mL, 1.875 pg/mL, and 9.375 pg/mL, respectively. The intra-assay coefficients (CV) for tested interleukins (IL-1β, IL-2, IL-4) were <10%.

#### 2.6.3. Intestinal Morphometry

To evaluate intestinal morphometry, ileal samples (n = 6 per treatment) were fixed in 10% neutral-buffered formalin, dehydrated in graded ethanol, cleared in xylene, and embedded in paraffin. Serial sections (5 µm) were cut using a rotary microtome and stained with hematoxylin and eosin (H&E) according to standard histological procedures [34].

Briefly, the ileum samples were first dehydrated through a graded ethanol series, cleared in xylene, and embedded in paraffin wax. Using a rotary microtome, serial sections of 5 µm thickness were carefully cut and mounted onto glass slides. The stained ileum sections were examined under a microscope (DP71, Olympus, Japan), attached to the transmitted light microscope (BX61, Olympus, Japan) using a 20× objective lens. For quantitative analysis of the morphological parameters, Olympus CellSens Entry software (version 1.6–1.17 range, Olympus, Japan) was utilized, following the approach described by Munyaka et al. (2012). This method allowed for precise measurement and assessment of the various intestinal features under study [35].

Villus length (VL) was measured as the distance from the crypt–villus junction to the tip of the villus along its longitudinal axis. Crypt depth (CD) was defined as the distance from the base of the crypt to the crypt–villus junction. The villus length to crypt depth ratio (VL/CD) was calculated as the ratio of these two measurements. Villus thickness (VT) was measured at the midpoint of the villus, perpendicular to its longitudinal axis. Only well-oriented and intact villus–crypt units were selected for analysis.

#### 2.6.4. Total Antioxidant Capacity

Non-hemolyzed blood plasma underwent a Total Antioxidant Capacity (TAC) assay (Abnova Corp., Taoyuan, Taiwan) according to the manufacturer’s instructions. The absorbance values of samples (n = 6 per treatment) and standards were measured at 570 nm in duplicate using Synergy HT Multi-Mode Microplate Reader (BioTek Instruments Inc., Winooski, VT, USA). Results for the standards were plotted graphically to determine the TAC concentration, which was expressed in micromolar Trolox equivalents.

#### 2.6.5. Statistical Analysis

All results are presented as mean ± SEM in tables and figures. Data organization and graphical editing were performed using Microsoft Office Excel (version 16.0). Statistical analyses were conducted using IBM SPSS Statistics version 29.0 (IBM Corp., Armonk, NY, USA). Significant differences are indicated by different letters in tables and figures.

Statistical tests were selected according to data distribution. One-way analysis of variance (ANOVA) was applied for normally distributed data (e.g., body weight and ileum morphological parameters including villus length, crypt depth, width, and related ratios). For non-normally distributed variables (e.g., relative spleen weight, body weight gain, weekly weight gain, interleukin concentrations (IL-1β, IL-2, IL-4), and total antioxidant capacity), the Kruskal–Wallis non-parametric test was used, followed by Dunn’s multiple comparison post hoc test with Bonferroni correction.

The level of statistical significance was set at *p* < 0.05. Assumptions of normality and homogeneity of variance were evaluated where applicable for parametric analyses. No observations were excluded as outliers, and all data points were retained to reflect biological variability among animals.

## 3. Results

### 3.1. Results for Body Weight, Body Weight Gain, and Weekly Body Weight Gain

At hatch (day 0), the average body weight of Japanese quail chicks was 8.73 ± 0.07 g (range: 7.2–9.8 g), which is consistent with reported hatch weight values for this species [36]. No significant differences in body weight were observed among selected 0-day chicks (*p* > 0.05).

**Figure 3:**
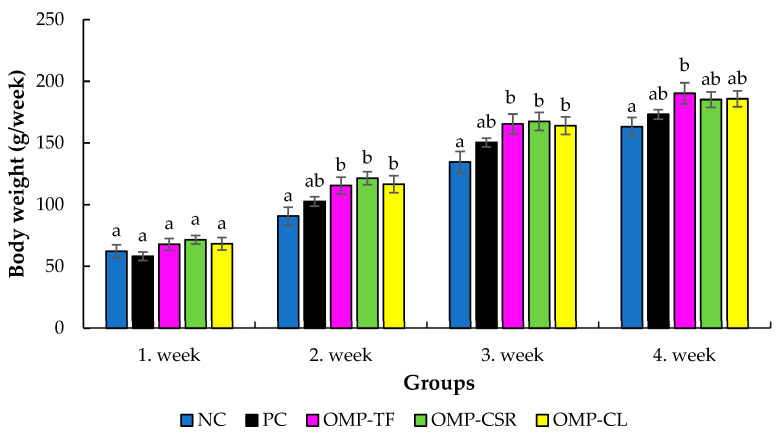
The effect of Oyster mushroom product supplementation on the average body weight of the male *C. japonica* birds during the trial. The results are MEAN ± SEM (body weight of male birds in g/each group). Individual male birds were used as the experimental unit for all growth performance analyses, with weekly measurements evaluated over the experimental period. Columns sharing a common letter within a week do not differ (*p* > 0.05) and are different (a, b), where data differed significantly (*p* < 0.05). The abbreviations are as follows: NC+SI: Fed with basal diet; NC+LPS: Fed with basal diet; PC+LPS: Fed with 0.1% commercially available β-glucan from *Saccharomyces cerevisiae* with 80 *w*/*w*% of β-glucan content; OMP-TF+LPS: 1% freeze-dried oyster mushroom powder from total fresh fruiting body; OMP-CSR+LPS: 1% freeze-dried oyster mushroom powder from cooked (90 °C, 4 h) solid residue; OMP-CL+LPS: 1% freeze-dried oyster mushroom powder from the cooked (90 °C, 4 h) liquid.

Figure 3 shows the effect of *P. ostreatus* supplementation on body weight in male *C. japonica*. No significant differences among groups were observed during week 1 (*p* > 0.05). However, significant differences emerged from week 2 onwards. In week 2, all mushroom-supplemented groups showed significantly higher body weight compared with the NC group (overall ANOVA: *p* = 0.007). A similar pattern was observed in week 3, where all supplemented groups again differed significantly from the NC group (*p* < 0.001 for all comparisons). In week 4, only the OMP-TF group maintained a significantly higher body weight compared with the NC group (*p* = 0.045), while no significant differences were detected for the remaining treatments.

**Figure 4:**
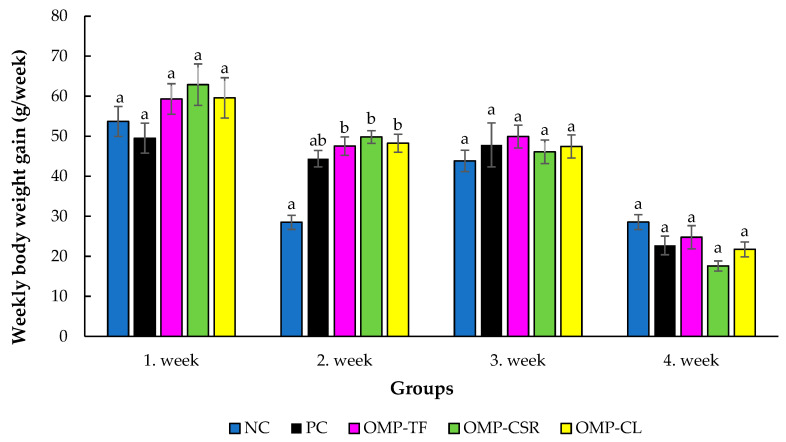
The effect of Oyster mushroom products on weekly body weight gain of male Japanese quails. The results are MEAN ± SEM (weekly body weight gains of male quails for 1, 2, 3, and 4 weeks). Individual male birds were used as the experimental unit for all growth performance analyses, with weekly measurements evaluated over the experimental period. Columns sharing a common letter within a week do not differ (*p* > 0.05) and are different (a, b), where data differed significantly (*p* < 0.05). The abbreviations are as follows: NC+SI: Fed with basal diet; NC+LPS: Fed with basal diet; PC+LPS: Fed with 0.1% commercially available β-glucan from *Saccharomyces cerevisiae* with 80 *w*/*w*% of β-glucan content; OMP-TF+LPS: 1% freeze-dried oyster mushroom powder from total fresh fruiting body; OMP-CSR+LPS: 1% freeze-dried oyster mushroom powder from cooked (90 °C, 4 h) solid residue; OMP-CL+LPS: 1% freeze-dried oyster mushroom powder from the cooked (90 °C, 4 h) liquid.

In weeks 1, 3, and 4, no significant differences were observed among the groups (*p* > 0.05) for weekly body weight gain. However, significant differences were detected in week 2 (*p* = 0.010). The negative control group (NC) showed significantly lower body weight gain compared with all oyster mushroom-supplemented groups: OMP-TF (*p* = 0.005), OMP-CSR (*p* = 0.002), and OMP-CL (*p* = 0.007), as can be seen in Figure 4.

**Figure 5:**
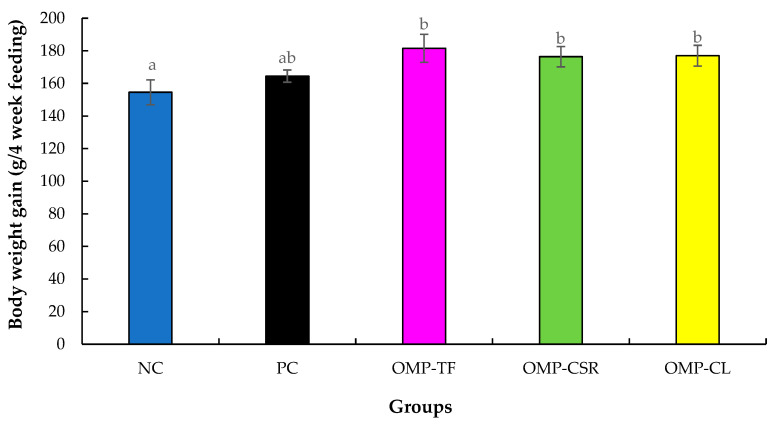
The effect of Oyster mushroom products on body weight gain of male Japanese quails. The results are MEAN ± SEM (body weight gains of male quails for 4 weeks (28 days). Individual male birds were used as the experimental unit for all growth performance analyses, with weekly measurements evaluated over the experimental period. Columns sharing a common letter within a week do not differ (*p* > 0.05) and are different (a, b), where data differed significantly (*p* < 0.05). The abbreviations are as follows: NC+SI: Fed with basal diet; NC+LPS: Fed with basal diet; PC+LPS: Fed with 0.1% commercially available β-glucan from *Saccharomyces cerevisiae* with 80 *w*/*w*% of β-glucan content; OMP-TF+LPS: 1% freeze-dried oyster mushroom powder from total fresh fruiting body; OMP-CSR+LPS: 1% freeze-dried oyster mushroom powder from cooked (90 °C, 4 h) solid residue; OMP-CL+LPS: 1% freeze-dried oyster mushroom powder from the cooked (90 °C, 4 h) liquid.

During the experimental period, significantly lower (*p* < 0.05) body weight gain was observed in the NC group compared with all of the oyster mushroom supplemented groups, like the OMP-TF (*p* = 0.013), OMP-CSR group (*p* = 0.040), and the OMP-CL group (*p* = 0.014), as presented in Figure 5.

### 3.2. Relative Spleen Weight

**Figure 6:**
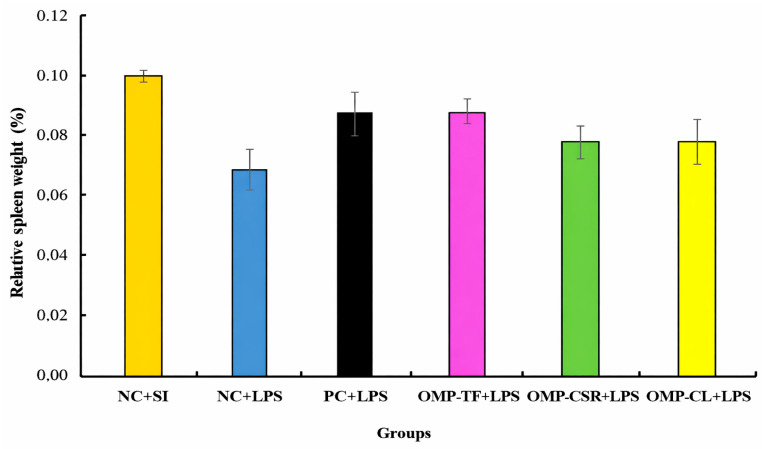
The effect of Oyster mushroom products and *Escherichia coli* LPS injection on male Japanese quails’ relative weight of the spleen. The results are MEAN ± SEM (Relative weight of spleen of male quails after 4-week (28-day) feeding period; n = 6 biological replicates per treatment (individual male birds)). Groups do not differ (*p* > 0.05). The abbreviations are as follows: NC+SI: Fed with basal diet; NC+LPS: Fed with basal diet; PC+LPS: Fed with 0.1% commercially available β-glucan from *Saccharomyces cerevisiae* with 80 *w*/*w*% of β-glucan content; OMP-TF+LPS: 1% freeze-dried oyster mushroom powder from total fresh fruiting body; OMP-CSR+LPS: 1% freeze-dried oyster mushroom powder from cooked (90 °C, 4 h) solid residue; OMP-CL+LPS: 1% freeze-dried oyster mushroom powder from the cooked (90 °C, 4 h) liquid. Negative control (NC+LPS), Positive control (PC+LPS), and mushroom supplementations (OMP-TF, OMP-CSR, OMP-CL) were injected with 1.5 mg (per kg body weight) O55:B5 serotype LPS (from *E. coli* cell wall), and NC-SI was injected with saline injection (1.5 mg/kg) to mitigate stress effects caused by LPS injection.

Figure 6 illustrates the effect of *P. ostreatus*-based supplementation on the spleen-to-body weight ratio in male *C. japonica.* One-way ANOVA revealed no statistically significant differences between treatment groups (*p* = 0.364). The largest numerical difference was observed between the NC+SI and NC+LPS groups; however, this difference did not reach statistical significance.

### 3.3. Effects of OMP Supplementation on Inflammatory Status Indicators and Immune Activity

**Figure 7:**
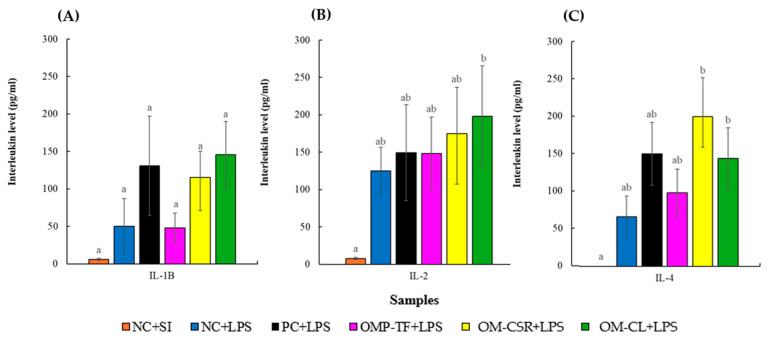
The effect of Oyster mushroom products and *Escherichia coli* LPS injection on male Japanese quails’ plasma cytokine levels (IL-1β (**A**), IL-2 (**B**), IL-4 (**C**)). The results are MEAN ± SEM (pg/mL for interleukin IL-1β, IL-2, IL-4 in blood plasma taken after 4-week (28-day) feeding period; n = 6 biological replicates per treatment (individual male birds)). Columns sharing a common letter within a week do not differ (*p* > 0.05) and are different (a, b), where data differed significantly (*p* < 0.05). The abbreviations are as follows: NC+SI: Fed with basal diet; NC+LPS: Fed with basal diet; PC+LPS: Fed with 0.1% commercially available β-glucan from *Saccharomyces cerevisiae* with 80 *w*/*w*% of β-glucan content; OMP-TF+LPS: 1% freeze-dried oyster mushroom powder from total fresh fruiting body; OMP-CSR+LPS: 1% freeze-dried oyster mushroom powder from cooked (90 °C, 4 h) solid residue; OMP-CL+LPS: 1% freeze-dried oyster mushroom powder from the cooked (90 °C, 4 h) liquid. Negative control (NC+LPS), Positive control (PC+LPS), and mushroom supplementations (OMP-TF, OMP-CSR, OMP-CL) were injected with 1.5 mg (per kg body weight) O55:B5 serotype LPS (from *E. coli* cell wall); furthermore, NC-SI was injected with saline injection (1.5 mg/kg) to mitigate stress effects caused by LPS injection.

The results of cytokine analysis, including interleukin (IL-1β, IL-2, and IL-4) concentrations, are presented separately in Figure 7A, Figure 7B, and Figure 7C, respectively. Each cytokine was analyzed independently to evaluate the specific effect of the treatments on the different immune pathways.

Although LPS administration resulted in markedly higher cytokine concentrations compared with the placebo control (NC+SI) at the descriptive level, substantial inter-individual variability within the LPS-treated groups limited the detection of statistically significant differences in pairwise comparisons. Consequently, none of the mushroom-supplemented LPS groups differed significantly from the LPS-treated control (NC+LPS) for IL-1β, IL-2, or IL-4 concentrations after correction for multiple testing.

No significant differences were observed in IL-1β concentrations among experimental groups (*p* = 0.057). In contrast, IL-2 concentrations differed significantly among groups (*p* = 0.029), with a significant pairwise difference detected only between the NC+SI and OMP+CL+LPS groups (adjusted *p* = 0.042).

Similarly, IL-4 levels showed significant overall group differences (*p* = 0.001). However, none of the treatment groups differed significantly from NC+LPS following multiple comparison correction. Although an increasing tendency was observed in the OMP+CSR+LPS group relative to NC+LPS in unadjusted comparisons, this difference did not remain significant after correction for multiple testing. Furthermore, the PC+LPS group did not differ significantly from NC+LPS or from any other treatment group.

### 3.4. Ileum Morphometry

**Figure 8:**
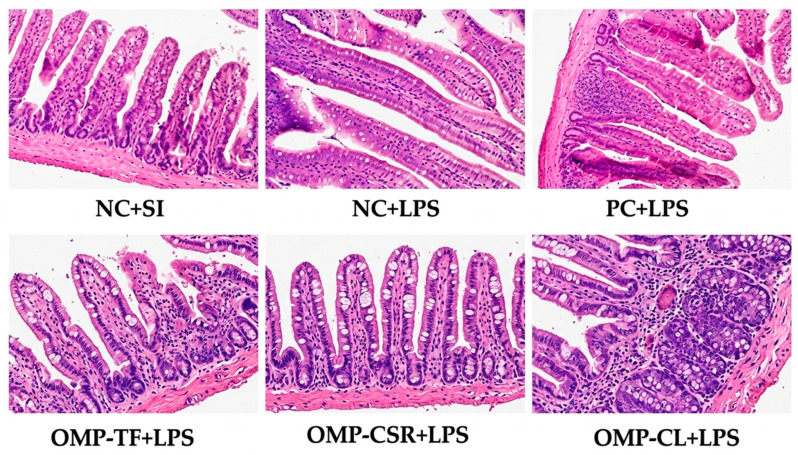
The effect of Oyster mushroom products and *Escherichia coli* LPS injection on male Japanese quails’ villus morphometry. The abbreviations are as follows: NC+SI: Fed with basal diet; NC+LPS: Fed with basal diet; PC+LPS: Fed with 0.1% commercially available β-glucan from *Saccharomyces cerevisiae* with 80 *w*/*w*% of β-glucan content; OMP-TF+LPS: 1% freeze-dried oyster mushroom powder from total fresh fruiting body; OMP-CSR+LPS: 1% freeze-dried oyster mushroom powder from cooked (90 °C, 4 h) solid residue; OMP-CL+LPS: 1% freeze-dried oyster mushroom powder from the cooked (90 °C, 4 h) liquid. Negative control (NC+LPS), Positive control (PC+LPS), and mushroom supplementations (OMP-TF, OMP-CSR, OMP-CL) were injected with 1.5 mg (per kg body weight) O55:B5 serotype LPS (from *E. coli* cell wall); furthermore, NC-SI was injected with saline injection (1.5 mg/kg) to mitigate stress effects caused by LPS injection.

We examined the morphological parameters of the intestinal villi in the following treatment groups: NC+SI, NC+LPS, PC+LPS, OMP-TF+LPS, OMP-CSR+LPS, and OMP-CL+LPS. Figure 8 illustrates the microscopic images of the ileum samples at 20× magnification.

Additionally, Table 3 presents the results of the villus morphological parameters (length, depth, width, and length-to-depth ratio). No significant differences were observed between villus length, depth, villus length/crypt depth ratio, total villus size, and villus width (*p* > 0.05).

### 3.5. Total Antioxidant Capacity in Blood Plasma

**Figure 9:**
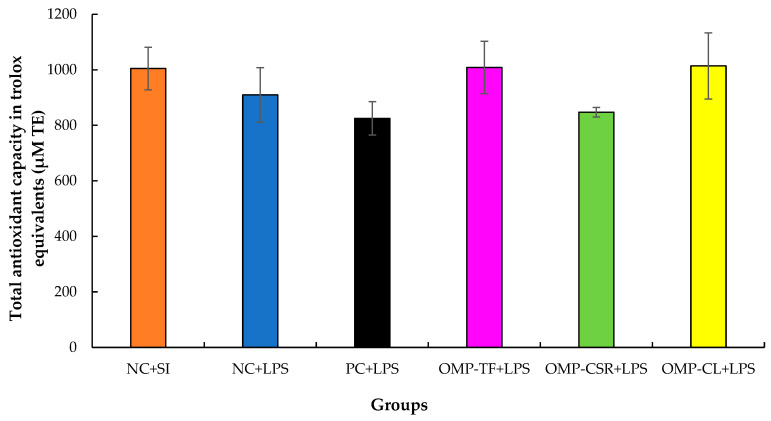
The effect of Oyster mushroom products and *Escherichia coli* LPS injection on male Japanese quails’ Total Antioxidant Capacity (TAC). The results are MEAN ± SEM (µM Trolox equivalents for Total Antioxidant Capacity in plasma taken after 4-week (28-day) feeding period; n = 6 biological replicates per treatment (individual male birds)). Groups do not differ (*p* > 0.05). The abbreviations are as follows: NC+SI: Fed with basal diet; NC+LPS: Fed with basal diet; PC+LPS: Fed with 0.1% commercially available β-glucan from *Saccharomyces cerevisiae* with 80 *w*/*w*% of β-glucan content; OMP-TF+LPS: 1% freeze-dried oyster mushroom powder from total fresh fruiting body; OMP-CSR+LPS: 1% freeze-dried oyster mushroom powder from cooked (90 °C, 4 h) solid residue; OMP-CL+LPS: 1% freeze-dried oyster mushroom powder from the cooked (90 °C, 4 h) liquid. Negative control (NC+LPS), Positive control (PC+LPS), and mushroom supplementations (OMP-TF, OMP-CSR, OMP-CL) were injected with 1.5 mg (per kg body weight) O55:B5 serotype LPS (from *E. coli* cell wall), and NC-SI was injected with saline injection (1.5 mg/kg) to mitigate stress effects caused by LPS injection.

Figure 9 presents the results of the effect of mushroom supplementation on total antioxidant capacity, expressed in Trolox equivalents (µM TE). No significant differences were observed among experimental groups according to the Kruskal–Wallis test (*p* = 0.281). Dunn’s post hoc pairwise comparisons with Bonferroni correction confirmed the absence of statistically significant differences between any treatment groups.

## 4. Discussion and Future Directions

The *Pleurotus ostreatus* mushroom investigated in this study is one of the most readily available mushrooms, ranking second in global cultivation after *Agaricus bisporus* [37]. Our study’s hypothesis centers on the mitigation capacity of *P. ostreatus*-derived supplementation on poultry’s immune function and overall health. Combining bioactive compounds, such as β-glucans, polyphenols, and other bioactive metabolites, may work together to modulate immune system activity more effectively than individual components. This synergy could enhance the immune system’s resilience, particularly under stress conditions like *E. coli* LPS injection. Lipopolysaccharide (LPS) is a key component of the outer membrane of Gram-negative bacteria and acts as a potent immune system activator. Exposure to LPS induces a cascade of immune system activities, including fever, inflammation, and the production of cytokines. These responses serve as indicators of immune function and stress resilience [38,39]. LPS from *E. coli* have been used to activate the immune system, enabling the evaluation of how *P. ostreatus*-based supplementation modulates these responses and potentially mitigates immunosuppressive effects. It should be noted that the present study evaluated dietary effects under inflammatory (LPS-challenged) conditions, and therefore results reflect immune-modulated rather than basal physiological responses. Our study aimed to demonstrate that using pre-cooked (total fresh mushroom, OMP-TF) and uncooked (separated solid residue (OMP-CSR) and liquid fraction (OMP-CL)) freeze-dried *P. ostreatus* mushroom powders as a feed additive supports the immune function and overall health of *C. japonica,* by valorizing economically valuable mushroom material with diverse bioactive compounds.

Numerous studies have shown that mushroom powder supplements, including those based on *P. ostreatus* and *A. bisporus*, positively influence poultry growth performance, such as weight gain and feed conversion [20,40,41]. The results of the present study indicate that *P. ostreatus* supplementation modulated growth performance in male Japanese quail under LPS-induced challenge conditions. The absence of significant differences at hatch and during the first week confirms the uniformity of the experimental groups and suggests that early post-hatch growth was not influenced by dietary treatments.

All mushroom-derived products enhanced body weight compared with the negative control during weeks 2 and 3, suggesting a protective or growth-supporting effect under inflammatory challenge conditions. Although this advantage decreased by week 4, the sustained effect observed in the OMP-TF group indicates that the total fruiting body preparation may provide longer-lasting benefits, potentially due to greater retention or bioavailability of bioactive compounds. Oyster mushroom supplementation modulated weekly body weight gain during week 2, suggesting a protective effect against LPS-induced growth suppression during a critical growth phase. The lack of differences in the other weeks indicates that this benefit was primarily transient and most evident during early development. *P. ostreatus* mushroom powders modulated overall weight gain, suggesting that mushroom-derived bioactive compounds may have supported nutrient utilization and reduced inflammation-associated growth suppression.

Overall, the combined body weight and weight gain responses indicate that *P. ostreatus* supplementation partially mitigated LPS-associated growth impairment, with the most pronounced effects observed during the rapid growth phase.

The beneficial effects can primarily be attributed to the β-glucan content, which supports immune function and promotes individual growth [42]. β-glucans act as immunomodulators, enhancing both innate and adaptive immune system activities in poultry, improving overall health, and achieving optimal growth performance [43]. The growth performance-enhancing effects of mushroom-based supplements can be linked to their ability to enhance gut health and the balanced composition of the gut microbiome, which is essential for optimal nutrient absorption and digestive efficiency, ultimately leading to higher weight gain in poultry [44]. Nevertheless, further investigations focusing on gut health, immune biomarkers, and nutrient digestibility are warranted to better elucidate the mechanisms underlying the observed performance-enhancing effects.

The measurement of spleen weight, often expressed as the relative weight of spleen, is a key indicator in studies focusing on immunity and health [45,46]. Spleen measurements were included in this study because the spleen plays a crucial role in immune system regulation and provides valuable insights into the physiological and immunological changes in birds [47]. Reducing the weight of immunity-related organs may indicate immunosuppression, whereas an increase typically reflects enhanced immune activity [48], while excessive enlargement of the spleen (splenomegaly) may also represent a pathological condition rather than a beneficial immune system activity. Splenomegaly can be associated with systemic inflammation, infection, endotoxin exposure, or immune overstimulation, and may indicate increased immune cell proliferation, congestion, or infiltration of inflammatory cells [49].

Our measured spleen-to-body weight range of 0.06–0.13% fits well within, or very close to, the published normal ranges for quail of similar age [50,51], suggesting that the experimental treatments did not result in abnormal spleen development. One-way ANOVA indicated no statistically significant differences between treatment groups (*p* = 0.364), and therefore no biological interpretation was made regarding differences among treatments. Although some numerical variation was observed between groups, particularly between NC+SI and NC+LPS, these differences were not statistically significant. It is also noted that LPS challenge may influence spleen weight depending on dose and duration; however, in the present short-term experimental setup, no measurable effect on spleen size was detected.

The cytokine responses observed following *E. coli* LPS challenge and mushroom-based feed supplementation were broadly consistent with previous studies describing the immunomodulatory properties of oyster mushroom (*P. ostreatus*) and other β-glucan-containing dietary sources. Earlier reports have demonstrated that mushroom-derived bioactive compounds can influence immune signaling pathways and cytokine regulation.

In the present study, IL-1β concentrations showed marked numerical variation among treatment groups; however, no statistically significant differences were detected following global non-parametric testing. This finding suggests that mushroom supplementation did not substantially modify the LPS-induced pro-inflammatory IL-1β response under the applied experimental conditions. Nevertheless, the elevated descriptive values observed in some mushroom-supplemented groups may indicate biological responsiveness consistent with previous reports showing that mushroom-derived β-glucans can stimulate pro-inflammatory cytokine production [52,53,54].

Similarly, IL-2 concentrations exhibited variability among treatments, although statistically significant pairwise differences were limited. While earlier studies have reported enhanced IL-2 production following mushroom supplementation and associated T-cell activation [55], the present results indicate that mushroom-derived products did not significantly alter the LPS-induced IL-2 response compared with the LPS-treated control group.

According to the literature, mushroom-based feed supplementation can enhance Th2-type immune system activities and IL-4 production, crucial in regulating allergic reactions and inflammatory responses [56]. In the present study, however, none of the mushroom-supplemented LPS groups showed statistically significant differences in IL-4 levels compared with the LPS-treated control (NC+LPS), suggesting that the treatments did not markedly modify the LPS-induced IL-4 response under the applied experimental conditions.

The evaluation of villus morphometry is paramount, particularly in the middle section (jejunum) and the small intestine’s last part (ileum). This facilitates nutrient digestion and absorption [57], emphasizing vitamins and nutrients synthesized in earlier sections. Measuring villus dimensions, such as length, depth, length-to-depth ratio, and width, is particularly useful in determining how the applied diet influences poultry health [2]. The measured ileum villus parameters in male Japanese quails ranged as follows: villus length varied between 452.16 and 555.83 µm, villus depth ranged from 149.11 to 175.21 µm, villus length-to-crypt depth ratio was 2.89–3.47, total villus length ranged from 603.93 to 731.04 µm, and villus width varied between 134.69 and 153.70 µm, which values are fully consistent with the villus morphometry of healthy 4-week-old Japanese quails, indicating that the villus structure of the studied birds can be considered physiologically normal [58].

Consistent with our findings, a study reported that a 1% oyster mushroom powder-based diet did not significantly affect (*p* > 0.05) ileal parameters, including average villus height and crypt depth [59]. Additionally, Toghyani et al. (2012) documented the ineffectiveness of mushroom powder on lymphoid organs, a finding supported by several researchers [60,61]. However, Mahfuz et al. (2020) observed that mushroom powders moderately modulated intestinal morphometry. They noted, however, that significant changes often require higher supplementation levels (%) or combined prebiotic formulations [62].

Total antioxidant capacity (TAC) measured in poultry blood plasma provides important information regarding systemic antioxidant defense and the ability to counteract oxidative stress [63]. In the present study, TAC values did not differ significantly among experimental groups, and mushroom-based dietary supplementation did not significantly modify antioxidant capacity compared with the LPS-treated control (NC+LPS). These findings suggest that the antioxidant response under the applied experimental conditions was not markedly influenced by dietary mushroom supplementation.

The findings of this study are directly applicable to male Japanese quails and likely generalize to other poultry species under similar rearing and dietary conditions. While the underlying immunomodulatory mechanisms of *P. ostreatus* bioactive compounds, such as β-glucans, may be relevant to other animals or humans, extrapolation to human biology should be made with caution, and further research is needed to confirm such effects.

It should be noted that the observed improvements in growth performance were not consistently accompanied by significant changes in intestinal morphometry, cytokine responses, or antioxidant capacity under the present experimental conditions. This suggests that the growth-promoting effects of *P. ostreatus* supplementation may be mediated through multifactorial and potentially subtle physiological mechanisms not fully captured by the selected biomarkers, including nutrient utilization efficiency and early-stage metabolic adaptations. Further targeted studies are therefore required to elucidate the precise mechanisms underlying these performance responses.

Future research should explore the large-scale application of *P. ostreatus* (oyster mushroom)-based diets in commercial poultry farming and optimize processing techniques to maximize β-glucan content. Investigating the synergistic interactions between the bioactive compounds in *P. ostreatus* and those in other wild and cultivated mushroom species is crucial for enhancing their potential benefits.

Several critical questions must be explored to fully grasp the potential of the diverse bioactive compounds derived from oyster mushrooms. Future research should emphasize this matrix’s composition and bioavailability, particularly concerning the effects of heat treatments, with a special focus on low-temperature, long-time cooking methods. Additionally, the potential role of carbon nanodots in enhancing the utilization of these bioactive compounds presents an exciting avenue for investigation.

## 5. Conclusions

This study evaluated the effects of a β-glucan-rich, freeze-dried *Pleurotus ostreatus*-based diet on growth performance and selected physiological parameters in 4-week-old male Japanese quails (*Coturnix japonica*) under *Escherichia coli* LPS challenge. Mushroom supplementation modulated growth performance, particularly during the rapid growth phase, as indicated by increased body weight and body weight gain compared with the non-supplemented control group. These findings suggest a partial mitigation of LPS-associated growth suppression. Although cytokine concentrations increased following LPS administration, mushroom supplementation did not significantly affect IL-1β, IL-2, or IL-4 levels compared with the LPS-treated control after multiple comparison correction. Similarly, spleen weight, ileum morphometry, and total antioxidant capacity were not significantly influenced by dietary treatments and remained within physiological ranges. Overall, the results indicate that *P. ostreatus* supplementation modulated growth performance under inflammatory challenge without measurable effects on the assessed immune or intestinal parameters. These findings suggest that the tested mushroom products may serve as potential growth-supporting feed additives under inflammatory challenge conditions; however, further studies are required to clarify their mechanisms of action and potential immunological effects. Nevertheless, the present study is limited by the lack of investigation into specific molecular mechanisms and feed efficiency. Future research should focus on evaluating feed efficiency alongside signaling pathways and barrier protein expression to better understand how different processing methods of *P. ostreatus* modulate intestinal integrity, nutrient utilization, and systemic immune system activity.

## Figures and Tables

**Table 1:** Experimental treatments and their descriptions.

Group Code	Description of Group	Type of Injection
**NC** **+** **SI**	The basal diet marked as a “placebo control”	Saline injection 12 h before termination
**NC+LPS**	Fed basal diet marked as a “negative control”	LPS injection 12 h before termination
**PC+LPS**	0.1% commercially available β-glucan from *Saccharomyces cerevisiae* with 80 *w*/*w*% of β-glucan content marked as a “positive control”,	LPS injection 12 h before termination
**OMP-TF+LPS**	1% freeze-dried oyster mushroom powder from the total fresh fruiting body marked as an “oyster mushroom-based supplementation”,	LPS injection 12 h before termination
**OMP-CSR+LPS**	1% freeze-dried oyster mushroom powder from cooked (90 °C, 4 h) solid residue marked as an “oyster mushroom-based supplementation”	LPS injection 12 h before termination
**OMP-CL+LPS**	1% freeze-dried oyster mushroom powder from the cooked (90 °C, 4 h) liquid marked as an “oyster mushroom-based supplementation”	LPS injection 12 h before termination

Abbreviations: NC, negative control; SI, saline injection; LPS, Lipopolysaccharides; PC, positive control; OMP, oyster mushroom powder; TF, total fruit; CSR, cooked solid residue; CL, cooked liquid.

**Table 2:** Composition of the Basal and Experimental Diets (% as-fed basis), formulated to meet or exceed NRC (1994) recommendations.

Feed Ingredients (%)	NC +SI	NC +LPS	PC +LPS	OMP-TF +LPS	OMP-CSR +LPS	OMP-CL +LPS
Corn	21.99	21.99	21.75	20.09	20.09	20.09
Wheat	30.00	30.00	30.00	30.00	30.00	30.00
Soybean meal (46% CP)	36.25	36.25	36.29	36.58	36.58	36.58
Fishmeal	5.00	5.00	5.00	5.00	5.00	5.00
Sunflower oil	4.43	4.43	4.50	5.01	5.01	5.01
Limestone	1.01	1.01	1.01	1.00	1.00	1.00
MCP	0.37	0.37	0.37	0.37	0.37	0.37
Salt	0.24	0.24	0.24	0.24	0.24	0.24
L-Met	0.10	0.10	0.10	0.10	0.10	0.10
L-Thr	0.11	0.11	0.11	0.11	0.11	0.11
β-glucan	-	-	0.125	-	-	-
OMP-TF	-	-	-	1.00	-	-
OMP-CSR	-	-	-	-	1.00	-
OMP-CL	-	-	-	-	-	1.00
Vitamin and mineral premix ^a^	0.50	0.50	0.50	0.50	0.50	0.50
Total	100	100	100	100	100	100
Nutrient Content (Calculated to meet or exceed NRC, 1994 recommendation)						
Nutrients	NC +SI	NC +LPS	PC +LPS	OMP-TF +LPS	OMP-CSR +LPS	OMP-CL +LPS
Metabolisable Energy (MJ/kg)	12.13	12.13	12.13	12.13	12.13	12.13
Crude Protein (%)	24.00	24.00	24.00	24.00	24.00	24.00
Lysine (%)	1.37	1.37	1.37	1.37	1.37	1.37
Methionine (%)	0.50	0.50	0.50	0.50	0.50	0.50
Threonine (%)	1.02	1.02	1.02	1.02	1.02	1.02
Tryptophan (%)	0.30	0.30	0.30	0.30	0.30	0.30
Leucine (%)	1.86	1.86	1.86	1.86	1.86	1.86
Isoleucine (%)	1.04	1.04	1.04	1.04	1.04	1.04
Arginine (%)	1.60	1.60	1.60	1.60	1.60	1.60
Leu/Ile Ratio	1.80	1.80	1.80	1.80	1.80	1.80
Calcium (%)	0.80	0.80	0.80	0.80	0.80	0.80
Phosphorus (%)	0.59	0.59	0.59	0.59	0.59	0.59
Non-phytate P (%)	0.30	0.30	0.30	0.30	0.30	0.30
Sodium (%)	0.15	0.15	0.15	0.15	0.15	0.15

^a^ 1 kg premix provided: 1,000,000 NE vitamin A, 200,000 NE vitamin D3, 4900 mg/kg vitamin E, 200 mg vitamin K3, 150 mg vitamin B1, 500 mg vitamin B2, 1200 mg Ca-d-Pantothenate, 400 mg vitamin B6, 2 mg vitamin B12, 11 mg biotin, 2502 mg niacin, 60 mg folic acid, 300,000 mg choline chloride, 13,200 mg Zn, 1920 mg Cu, 9612 mg Fe, 13,200 mg Mn, 180 mg I, 42 mg Se, 12 mg Co.

**Table 3:** The effect of Oyster mushroom products and *Escherichia coli* LPS challenge on male Japanese quails’ villus morphological parameters (ileum length, depth, width, and ratios in (µm)).

Group	Villus Length	Villus Depth	Villus Length-to-Crypt Depth Ratio	Total Villus	Villus Width
**NC+SI**	489.28 ± 56.66	171.41 ± 12.23	2.89 ± 0.21	660.70 ± 66.45	153.70 ± 9.44
**NC+LPS**	452.16 ± 28.02	151.77 ± 6.22	3.04 ± 0.07	603.93 ± 34.13	136.80 ± 9.96
**PC+LPS**	555.83 ± 34.38	175.21 ± 11.28	3.33 ± 0.27	731.04 ± 38.01	149.87 ± 6.91
**OMP-TF+LPS**	492.74 ± 45.85	151.63 ± 5.07	3.35 ± 0.28	644.36 ± 47.91	137.05 ± 6.49
**OMP-CSR+LPS**	530.58 ± 44.51	173.38 ± 13.91	3.19 ± 0.25	703.96 ± 54.30	148.76 ± 6.90
**OMP-CL+LPS**	503.52 ± 46.00	149.11 ± 5.68	3.47 ± 0.29	652.63 ± 48.44	134.69 ± 6.58
***p*** **-value**	0.679	0.161	0.650	0.546	0.347

The results are MEAN ± SEM (µm) for villus morphological parameters (ileum length, depth, width, and ratios in (µm)) taken after 4-week (28-day) feeding period; n = 6 biological replicates per treatment (individual male birds)). Groups do not differ (*p* > 0.05). The abbreviations are as follows: NC+SI: Fed with basal diet; NC+LPS: Fed with basal diet; PC+LPS: Fed with 0.1% commercially available β-glucan from *Saccharomyces cerevisiae* with 80 *w*/*w*% of β-glucan content; OMP-TF+LPS: 1% freeze-dried oyster mushroom powder from total fresh fruiting body; OMP-CSR+LPS: 1% freeze-dried oyster mushroom powder from cooked (90 °C, 4 h) solid residue; OMP-CL+LPS: 1% freeze-dried oyster mushroom powder from the cooked (90 °C, 4 h) liquid. Negative control (NC+LPS), Positive control (PC+LPS), and mushroom supplementations (OMP-TF, OMP-CSR, OMP-CL) were injected with 1.5 mg (per kg body weight) O55:B5 serotype LPS (from *E. coli* cell wall); furthermore, NC-SI was injected with saline injection (1.5 mg/kg) to mitigate stress effects caused by LPS injection.

## Data Availability

The original contributions presented in this study are included in the article. Further inquiries can be directed to the corresponding author(s).
